# Targeted-release budesonide versus systemic corticosteroids in IgA nephropathy: a real-world comparative study

**DOI:** 10.3389/fimmu.2026.1831633

**Published:** 2026-07-29

**Authors:** Tianjiao Cui, Huibin Wu, Wenjian Zhu, Jiawen Lin, Shuping Li, Yuan Sui, Xuelin Zeng, Zhihua Zheng, Mingcheng Huang

**Affiliations:** 1 Department of Nephrology, Center of Kidney and Urology, The Seventh Affiliated Hospital, Sun Yat-Sen University, Shenzhen, Guangdong, China; 2 Department of Physiology, University of Oklahoma Health Sciences Center, Oklahoma City, OK, United States; 3 Molecular and Cellular Biology Laboratory, The Salk Institute for Biological Studies, La Jolla, CA, United States; 4 Department of Pharmacy, The Seventh Affiliated Hospital of Sun Yat-Sen University, Shenzhen, Guangdong, China

**Keywords:** budesonide, IgA nephropathy, proteinuria, renal protection, systemic corticosteroids

## Abstract

**Background:**

Targeted-release budesonide (Nefecon) has demonstrated efficacy in randomized trials for IgA nephropathy (IgAN), yet direct comparative real-world evidence against systemic corticosteroids is scarce, limiting clinical decision-making.

**Methods:**

We conducted a longitudinal real-world study of adult patients with biopsy-proven IgAN treated with either Nefecon or systemic corticosteroids. Renal outcomes, including estimated glomerular filtration rate (eGFR), urinary albumin-to-creatinine ratio (UACR), 24-hour urinary protein excretion (24hUP), and urinary red blood cell count (URBC), were assessed at baseline and at 3, 6, and 9 months. Longitudinal associations were evaluated using linear mixed-effects models adjusted for age, sex, systolic and diastolic blood pressure, with multiple imputation for missing data.

**Results:**

A total of 82 patients were included (Nefecon, n=32; systemic corticosteroids, n=50). Both treatments were associated with significant reductions in proteinuria-related outcomes over time. Compared with systemic corticosteroids, Nefecon showed a trend toward greater early reduction in UACR at 3 months after expanded adjustment and an adjusted exploratory short-term favorable difference in eGFR at 6 months. However, this eGFR difference was not sustained at 9 months, and the primary outcome (24hUP) showed no significant between-group difference at any time point.Severe infections and steroid-related adverse events were numerically less frequent in the Nefecon group.

**Conclusions:**

In routine clinical practice, Nefecon showed antiproteinuric efficacy comparable to systemic corticosteroids, with no significant difference in the primary outcome (24hUP) between groups. An exploratory short-term favorable eGFR signal was observed at 6 months, but this was not sustained at 9 months and should not be interpreted as evidence of sustained renal protection. Severe infections and steroid-related adverse events were numerically less frequent in the Nefecon group, but limited event counts warrant cautious interpretation. Longer follow-up is required to determine whether these exploratory findings are clinically meaningful.

## 1 Introduction

Immunoglobulin A nephropathy (IgA nephropathy, IgAN) is the most common primary glomerulonephritis worldwide and a leading cause of chronic kidney disease (CKD) and end-stage kidney disease (ESKD) (1). Despite its high prevalence, therapeutic options remain limited, and disease progression is associated with significant morbidity, cardiovascular risk, and socioeconomic burden. Current management of IgAN relies primarily on supportive care, including optimized blood pressure control, renin-angiotensin system (RAS) blockade, and lifestyle modifications (2). More recently, sodium-glucose cotransporter 2 (SGLT2) inhibitors have been incorporated into the KDIGO 2021 Clinical Practice Guideline for the Management of Glomerular Diseases based on evidence of renal and cardiovascular benefit in patients with IgAN (2). However, renal response to supportive therapy alone is often suboptimal, with complete remission rates as low as 6.3% at 3 months and 31.3% at 6 months in some cohorts (3). For patients with persistent proteinuria >0.75–1.0 g/day despite optimized supportive therapy, systemic corticosteroids have been used to slow disease progression. Nevertheless, their long-term efficacy remains controversial, and their use is frequently limited by substantial adverse effects, including infection, metabolic disturbances, weight gain, glucose intolerance, and bone toxicity (4). Thus, there is an urgent need for safer and more effective therapies that can modify the course of IgAN while minimizing systemic toxicity.

Recent advances in the understanding of IgAN pathogenesis highlight the role of mucosal immunity, particularly gut-associated lymphoid tissue (GALT), in the production of galactose-deficient IgA1 (Gd-IgA1), a key driver of immune complex formation and renal injury (5). This mechanistic insight has led to the development of targeted-release budesonide(Nefecon), an oral formulation designed to deliver budesonide-a potent glucocorticoid with high first-pass metabolism-directly to the distal ileum. By locally modulating mucosal B-cell activity and reducing pathogenic IgA production, Nefecon aims to treat IgAN at its source while minimizing systemic corticosteroid exposure (5). Randomized controlled trials, including the global phase III NefIgArd study, have demonstrated that Nefecon significantly reduces proteinuria and slows the decline in estimated glomerular filtration rate (eGFR) in patients with IgAN at high risk of progression (5). In Chinese subpopulations, Nefecon showed even greater preservation of eGFR and reduction in proteinuria compared to placebo, with a favorable safety profile consistent with its targeted mechanism of action (6).

In recent years, the therapeutic landscape for IgA nephropathy has evolved substantially. Multiple novel agents targeting distinct pathogenic pathways have entered clinical development or received regulatory approvals. These include APRIL inhibitors (e.g., sibeprenlimab), complement inhibitors targeting factor B (e.g., iptacopan) or C5 (e.g., ravulizumab), dual endothelin/angiotensin receptor antagonists (e.g., sparsentan), and selective endothelin receptor antagonists (e.g., atrasentan). Among these emerging therapies, targeted-release budesonide (Nefecon) is the first agent specifically designed to modulate gut-associated mucosal immunity-an upstream driver of pathogenic IgA production. This evolving landscape offers multiple opportunities for personalized treatment approaches in IgAN. However, randomized trials are conducted under tightly controlled conditions and may not fully reflect real-world clinical practice, where patients often exhibit greater heterogeneity in disease severity, comorbidities, concomitant medications, and treatment adherence.

To date, direct comparative evidence between Nefecon and systemic corticosteroids in real-world settings remains scarce. Such studies are essential to evaluate the effectiveness, safety, and practical applicability of Nefecon in routine clinical care, and to inform treatment decisions for clinicians managing patients with IgAN.

In this context, we conducted a longitudinal real-world study to compare the renal outcomes and safety profiles of targeted-release budesonide versus systemic corticosteroids in patients with IgAN. Using mixed-effects modeling and sensitivity analyses, we aimed to assess trajectories of eGFR, proteinuria (UACR and 24-hour urine protein), and hematuria (URBC) over a 9-month follow-up period, while comprehensively documenting adverse events. We hypothesized that Nefecon would provide similar or superior antiproteinuric efficacy with a more favorable safety profile compared to systemic corticosteroids in a real-world IgAN population. Our study seeks to complement randomized trial data and provide evidence to guide personalized therapeutic strategies in IgAN management.

## 2 Methods

### 2.1 Study design and participants

This was a single-center, longitudinal observational real-world study evaluating the effectiveness of targeted-release budesonide and systemic corticosteroids in the treatment of IgA nephropathy, conducted at The Seventh Affiliated Hospital of Sun Yat-sen University. The exclusion criteria included secondary IgAN, missing baseline or follow-up data, pregnancy or lactation, complicated with Henoch-Schönlein nephritis, chronic hepatic disease, malignant tumor, systemic lupus erythematosus or other connective tissue diseases, receipt of intravenous methylprednisolone pulses, and other conditions that might interfere with treatment or outcome assessment. This was a longitudinal observational study evaluating the effects of Nefecon(16 mg/d) compared with systemic corticosteroids (control group) (patients used prednisone or prednisolone (0.5–1 mg/kg/d; maximum, 60 mg/d) for 2 months, which was tapered to 5 mg every 2 weeks and stopped within 6 to 12 months) on renal-related biomarkers over time. Participants were followed from baseline through 3, 6, and 9 months after treatment initiation. The study population included adult patients with repeated measurements of renal function and proteinuria-related indicators during the follow-up period. Participants were classified into two groups according to treatment exposure: the Nefecon group and systemic corticosteroids (control group). All analyses were conducted at the individual level, accounting for within-subject correlations arising from repeated measurements. In both groups, background therapy with RAS blockers and SGLT2 inhibitors was maintained at stable, optimized, maximally tolerated doses according to KDIGO guidelines. Patients in both treatment groups were recruited and treated concurrently between October 2024 and October 2025. There was no temporal difference in treatment allocation between groups.

### 2.2 Outcomes

The study follow-up period was 9 months. The following baseline characteristics were assessed: age, sex, mean arterial pressure (MAP), 24-h total urinary protein excretion, serum creatinine level (Scr), and eGFR. MAP was calculated as diastolic blood pressure plus one-third of the pulse pressure. The eGFR was calculated by using the Chronic Kidney Disease Epidemiology Collaboration (CKD-EPI) creatinine equation. The “baseline” was defined as the time at which patients started treatment with Nefecon or systemic corticosteroids. These data were collected every 1–3 months during the follow-up. All assays were performed at a laboratory in the Department of Clinical Laboratory of the The Seventh Affiliated Hospital of Sun Yat -Sen University using standard methods.

Primary outcome: 24-hour urinary protein excretion (24hUP) was prespecified as the primary outcome. Secondary outcomes: urinary albumin-to-creatinine ratio (UACR), estimated glomerular filtration rate (eGFR), and serum albumin (ALB). Exploratory outcome: urinary red blood cell count (URBC).

Measurements were obtained at baseline and at 3, 6, and 9 months. Due to right-skewed distributions, 24hUP and URBC were log-transformed prior to modeling. For interpretability, estimates for log-transformed outcomes were exponentiated and presented as ratios.

### 2.3 Covariates

The primary multivariable model was adjusted for baseline demographic and clinical covariates, including sex, age, systolic blood pressure (SBP), and diastolic blood pressure (DBP). An expanded sensitivity model additionally adjusted for baseline eGFR, baseline log-transformed 24-hour urinary protein excretion (24hUP), disease duration, CKD stage, Oxford MEST-C pathological variables, and baseline use of ARB, SGLT2 inhibitors, and mineralocorticoid receptor antagonists (MRA). **Table 1** presents the results of the expanded model as the main reported analysis.

**Table 1:** Fixed effects from linear mixed-effects models.

Outcome	Effect	β (95% CI)	Ratio (95% CI)	*P*
eGFR	Treatment (Nefecon vs Control)	-2.607(-13.900, 8.685)	—	0.651
	Time: 3m vs Baseline	-12.078(-19.849, -4.306)	—	0.002
	Time: 6m vs Baseline	-9.083(-16.751, -1.415)	—	0.02
	Time: 9m vs Baseline	-15.090(-23.055, -7.126)	—	<0.001
	Treatment × 3m	11.670(-1.336, 24.675)	—	0.079
	Treatment × 6m	13.618(0.980, 26.256)	—	0.035
	Treatment × 9m	9.637(-3.069, 22.344)	—	0.137
UACR (log)	Treatment (Nefecon vs Control)	-0.348(-1.171, 0.475)	0.706(0.310, 1.608)	0.407
	Time: 3m vs Baseline	-0.438(-0.828, -0.048)	0.646(0.437, 0.954)	0.028
	Time: 6m vs Baseline	-1.177(-1.565, -0.790)	0.308(0.209, 0.454)	<0.001
	Time: 9m vs Baseline	-1.558(-1.970, -1.145)	0.211(0.139, 0.318)	<0.001
	Treatment × 3m	-0.606(-1.267, 0.054)	0.545(0.282, 1.056)	0.072
	Treatment × 6m	0.142(-0.518, 0.802)	1.152(0.596, 2.230)	0.674
	Treatment × 9m	0.158(-0.586, 0.902)	1.171(0.557, 2.464)	0.676
24hUP (log)	Treatment (Nefecon vs Control)	-0.215(-0.819, 0.390)	0.807(0.441, 1.477)	0.486
	Time: 3m vs Baseline	-0.654(-1.069, -0.240)	0.520(0.343, 0.787)	0.002
	Time: 6m vs Baseline	-0.952(-1.337, -0.566)	0.386(0.263, 0.568)	<0.001
	Time: 9m vs Baseline	-1.391(-1.802, -0.980)	0.249(0.165, 0.375)	<0.001
	Treatment × 3m	-0.316(-1.005, 0.373)	0.729(0.366, 1.452)	0.368
	Treatment × 6m	-0.221(-0.863, 0.421)	0.802(0.422, 1.524)	0.5
	Treatment × 9m	0.058(-0.689, 0.806)	1.060(0.502, 2.238)	0.878
URBC (log)	Treatment (Nefecon vs Control)	-0.626(-1.556, 0.305)	0.535(0.211, 1.357)	0.188
	Time: 3m vs Baseline	-0.447(-1.244, 0.350)	0.639(0.288, 1.419)	0.271
	Time: 6m vs Baseline	0.331(-0.435, 1.098)	1.393(0.647, 2.999)	0.396
	Time: 9m vs Baseline	-1.107(-1.851, -0.364)	0.330(0.157, 0.695)	0.004
	Treatment × 3m	0.482(-0.721, 1.684)	1.619(0.486, 5.388)	0.432
	Treatment × 6m	-0.876(-2.062, 0.310)	0.416(0.127, 1.363)	0.147
	Treatment × 9m	0.197(-1.012, 1.406)	1.218(0.364, 4.079)	0.749

Models included fixed effects for treatment group, time, their interaction, baseline eGFR, baseline log-transformed 24hUP, disease duration, CKD stage, Oxford MEST-C score, baseline ARB/SGLT2 inhibitor/MRA use, sex, age, SBP, and DBP, with a random intercept for each participant. 24hUP and URBC were log-transformed before modeling.

### 2.4 Statistical analysis

#### 2.4.1 Handling of missing data

Missing data in outcomes and covariates were handled using multiple imputation by chained equations (MICE). Imputation was conducted under the missing-at-random (MAR) assumption. Continuous variables were imputed using predictive mean matching to preserve the original data distribution and avoid implausible values.

Treatment group, sex, and participant identifiers were not imputed, but were included as predictors in the imputation models to improve the plausibility of the MAR assumption. All variables included in the analytical models were incorporated into the imputation process.

A total of 50 imputed datasets were generated. Primary analyses were performed separately within each imputed dataset, and parameter estimates and standard errors were combined using Rubin’s rules to obtain pooled effect estimates, 95% confidence intervals, and corresponding p-values.

The proportion and pattern of missing data were summarized before imputation. Missingness was mainly observed in repeated follow-up laboratory measurements, whereas treatment group and core baseline demographic variables were complete or nearly complete. Multiple imputation by chained equations was performed under the missing-at-random assumption. The imputation model included treatment group, participant identifier, demographic variables, baseline renal function, proteinuria-related variables, Oxford MEST-C pathological variables, supportive therapy variables, and repeated renal outcome measurements. Treatment group, sex, and participant identifiers were not imputed but were included as predictors where appropriate. A total of 50 imputed datasets were generated, and estimates were pooled using Rubin’s rules. Complete-case analyses were performed as sensitivity analyses to assess the influence of missing data handling.

#### 2.4.2 Descriptive analysis

Baseline characteristics and follow-up measurements were summarized using descriptive statistics. Continuous variables were described as median with interquartile range (IQR), and categorical variables were presented as counts with corresponding percentages.

For each outcome variable, the percentage change from baseline was calculated at 3, 6, and 9 months to characterize longitudinal trends over time. Changes were calculated within individuals relative to their baseline values.

In addition, subgroup analyses were performed according to baseline renal function. Participants were stratified into two subgroups using an estimated glomerular filtration rate (eGFR) threshold of 30 mL/min/1.73 m². Within each subgroup, percentage changes in outcomes at each follow-up time point were calculated to explore potential heterogeneity of treatment effects across levels of baseline kidney function.

#### 2.4.3 Linear mixed-effects models for primary analysis

The primary analysis was conducted using linear mixed-effects models (LMMs) to assess the longitudinal association between Nefecon group and systemic corticosteroids group outcome trajectories over time. Models included fixed effects for Nefecon group, time, and their interaction, as well as covariates (sex, age, SBP, DBP). This primary model included only sex, age, SBP, and DBP as covariates. An expanded sensitivity model additionally adjusted for baseline eGFR, baseline log-transformed 24hUP, disease duration, CKD stage, Oxford MEST-C variables, and baseline use of ARB, SGLT2 inhibitors, and MRA. The results of the expanded model are presented in **Table 1**. A random intercept for each participant was included to account for within-subject correlation.

The general form of the model was:


$${\text{Y}}_{\text{ij}}=\beta_{0}+\beta_{1}{\text{Group}}_{\text{i}}+\beta_{2}{\text{Time}}_{\text{ij}}+\beta_{3}({\text{Group}}_{\text{i}}\times {\text{Time}}_{\text{ij}})+{\text{Z}}_{\text{i}}\gamma +{\text{b}}_{\text{i}}+\epsilon_{\text{ij}}$$

where Y_ij_ denotes the outcome for individual i at time j, Z_i_ represents covariates, b_i_ is the subject-specific random intercept, and ϵ_ij_ is the residual error.

The interaction term between Nefecon group and time was the primary parameter of interest, representing the differential change from baseline between the Nefecon and Control groups at each follow-up time point.

#### 2.4.4 Sensitivity analyses

Specifically, in addition to the random-intercept linear mixed-effects models used in the primary analysis, alternative models incorporating subject-specific random slopes for time were fitted using the same multiply imputed datasets. This approach allowed individual participants to have distinct longitudinal trajectories over follow-up.

All sensitivity models included fixed effects for treatment group, time, and their interaction, as well as the same set of covariates (sex, age, systolic blood pressure, and diastolic blood pressure). Time was treated as a categorical variable, with baseline as the reference category, and treatment-by-time interaction terms quantified group differences in changes from baseline at each follow-up time point. Models were fitted using maximum likelihood, and results across imputations were pooled according to Rubin’s rules.

### 2.5 Statistical software

All analyses were performed using R (version 4.5.2). Linear mixed-effects models were fitted using the lme4 and lmerTest packages. Multiple imputation was conducted using the mice package. GEE analyses were performed using the geepack package. A two-sided *p*-value< 0.05 was considered statistically significant.

## 3 Results

### 3.1 Baseline characteristics and descriptive changes over follow-up

A patient flow diagram summarizing screening, exclusions, treatment allocation, and follow-up completion is presented in Figure S. A total of 82 adult patients with biopsy-proven IgA nephropathy were included in this study, comprising 50 patients treated with systemic corticosteroids and 32 patients treated with Nefecon. Baseline demographic characteristics were generally similar between the two groups, although several clinically relevant differences were observed (**Table 2**).

**Table 2:** Baseline characteristics of the study participants.

Variable	Level	Overall	Control	Nefecon	*P*
n		82	50	32	
Sex, n (%)	0	24 (29.3)	18 (36.0)	6 (18.8)	0.154
	1	58 (70.7)	32 (64.0)	26 (81.2)	
Age, median (IQR)		38.50 (32.00, 45.00)	37.50 (30.25, 45.75)	38.50 (33.00, 44.25)	0.732
SBP, median (IQR)		122.00 (112.00, 131.00)	123.00 (110.00, 130.75)	122.00 (115.00, 130.00)	0.586
DBP, median (IQR)		76.00 (71.00, 85.00)	77.00 (68.00, 85.00)	76.00 (73.50, 81.50)	0.831
Disease duration, median (IQR), months		48.00 (31.00, 72.00)	48.00 (37.25, 72.00)	38.00 (15.75, 73.50)	0.154
Cumulative dose, median (IQR), g		8.52 (4.88, 18.25)	6.65 (4.00, 12.30)	18.24 (7.56, 35.28)	<0.001
CKD stage, n (%)	1	18 (22.0)	12 (24.0)	6 (18.8)	0.856
	2	23 (28.0)	15 (30.0)	8 (25.0)	
	3	18 (22.0)	9 (18.0)	9 (28.1)	
	4	5 (6.1)	3 (6.0)	2 (6.2)	
	5	18 (22.0)	11 (22.0)	7 (21.9)	
Oxford M, n (%)	0	10 (13.7)	9 (18.0)	1 (4.3)	0.226
	1	63 (86.3)	41 (82.0)	22 (95.7)	
Oxford E, n (%)	0	55 (75.3)	36 (72.0)	19 (82.6)	0.494
	1	18 (24.7)	14 (28.0)	4 (17.4)	
Oxford S, n (%)	0	25 (34.2)	20 (40.0)	5 (21.7)	0.207
	1	48 (65.8)	30 (60.0)	18 (78.3)	
Oxford T, n (%)	0	41 (56.2)	31 (62.0)	10 (43.5)	0.333
	1	22 (30.1)	13 (26.0)	9 (39.1)	
	2	10 (13.7)	6 (12.0)	4 (17.4)	
Oxford C, n (%)	0	28 (38.4)	14 (28.0)	14 (60.9)	0.016
	1	40 (54.8)	31 (62.0)	9 (39.1)	
	2	5 (6.8)	5 (10.0)	0 (0.0)	
Baseline ARB use, n (%)	0	52 (66.7)	43 (86.0)	9 (32.1)	<0.001
	1	26 (33.3)	7 (14.0)	19 (67.9)	
Baseline SGLT2 inhibitor use, n (%)	0	67 (85.9)	49 (98.0)	18 (64.3)	<0.001
	1	11 (14.1)	1 (2.0)	10 (35.7)	
Baseline MRA use, n (%)	0	76 (97.4)	50 (100.0)	26 (92.9)	0.243
	1	2 (2.6)	0 (0.0)	2 (7.1)	
ARB use during treatment, n (%)	0	24 (29.3)	11 (22.0)	13 (40.6)	0.119
	1	58 (70.7)	39 (78.0)	19 (59.4)	
SGLT2 inhibitor use during treatment, n (%)	0	70 (85.4)	44 (88.0)	26 (81.2)	0.601
	1	12 (14.6)	6 (12.0)	6 (18.8)	
MRA use during treatment, n (%)	0	79 (96.3)	49 (98.0)	30 (93.8)	0.691
	1	3 (3.7)	1 (2.0)	2 (6.2)	
uacr, median (IQR)		676(288, 1196)	714(320, 1327)	609(260, 961)	0.256
24hUP, median (IQR)		1407(782, 2642)	1349(773, 3487)	1465(867, 2585)	0.867
URBC, median (IQR)		37 (17, 98)	51(22, 137)	24 (5, 63)	0.041
egfr, median (IQR)		78 (48, 106)	86(61, 108)	59(46, 86)	0.075

Continuous variables are presented as median (IQR), and categorical variables are presented as n (%). P values compare the Control and Nefecon groups. SBP, systolic blood pressure; DBP, diastolic blood pressure; ARB, angiotensin receptor blocker; SGLT2, sodium-glucose cotransporter 2; MRA, mineralocorticoid receptor antagonist; CKD, chronic kidney disease.

The proportion of male patients was numerically higher in the Nefecon group than in the systemic corticosteroid group (81.2% vs. 64.0%), but this difference did not reach statistical significance (*P* = 0.154). Median age was identical between groups (38.50 years in the Nefecon group vs. 37.50 years in the systemic corticosteroid group, *P* = 0.732), and baseline systolic and diastolic blood pressure levels were also comparable. Disease duration was numerically shorter in the Nefecon group (38.00 months (IQR, 15.75–73.50)) than in the systemic corticosteroid group (48.00 months (IQR, 37.25–72.00)), although the difference was not statistically significant (*P* = 0.154). In contrast, cumulative treatment dose was significantly higher in the Nefecon group than in the systemic corticosteroid group (18.24 g (IQR, 7.56–35.28) vs. 6.65 g (IQR, 4.00–12.30), *P* < 0.001). Baseline renal outcome measures, including eGFR, 24hUP, UACR, and URBC, are summarized in **Table 2** and were further accounted for in the longitudinal mixed-effects models where appropriate.

Regarding pathological characteristics, CKD stage and most Oxford MEST variables were not significantly different between groups. However, Oxford C score differed significantly between groups (*P* = 0.016), with a higher proportion of Oxford C0 in the Nefecon group and a higher proportion of Oxford C1–C2 in the systemic corticosteroid group. Baseline nephroprotective therapy uses also differed between groups. Baseline ARB use was more frequent in the Nefecon group than in the systemic corticosteroid group (67.9% vs. 14.0%, *P* < 0.001), as was baseline SGLT2 inhibitor use (35.7% vs. 2.0%, *P* < 0.001). Baseline MRA use was uncommon and did not differ significantly between groups. During treatment, the use of ARB, SGLT2 inhibitors, and MRA was not significantly different between groups.

**Figure 1:**
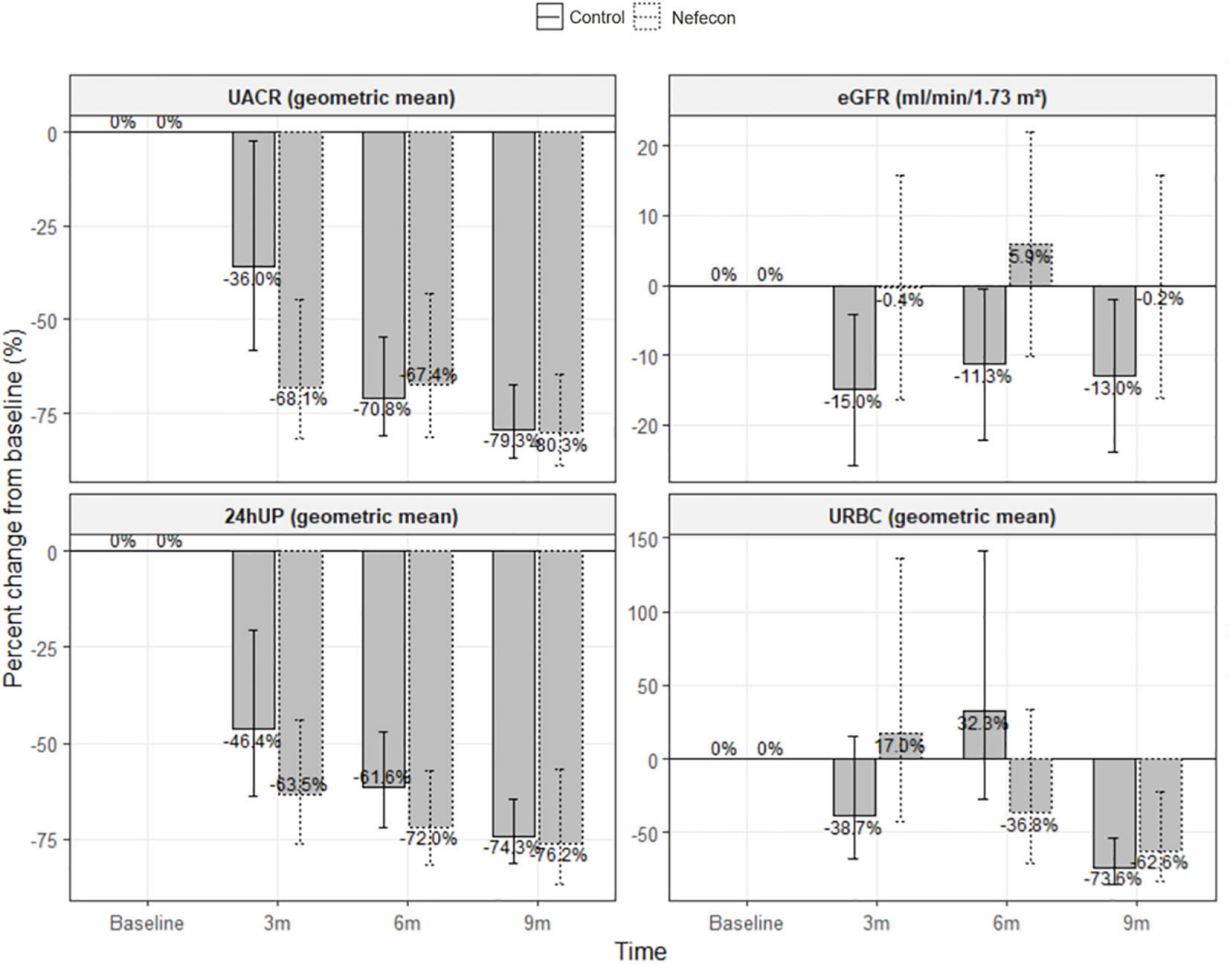
Descriptive changes of the study participants.

During follow-up at 3, 6, and 9 months, proteinuria-related outcomes showed a general downward trend over time. At 3 months, the Nefecon group exhibited a lower UACR compared with the systemic corticosteroid group (155.00 (77.00, 494.75) vs. 396.50 (122.00, 1266.75), *P* = 0.013). URBC was also lower in the Nefecon group at 6 months (P = 0.012). No other between-group differences at individual follow-up time points reached statistical significance. Percentage changes in renal outcomes relative to baseline are shown in **Figure 1**.

### 3.2 Longitudinal mixed-effects analysis

**Figure 2:**
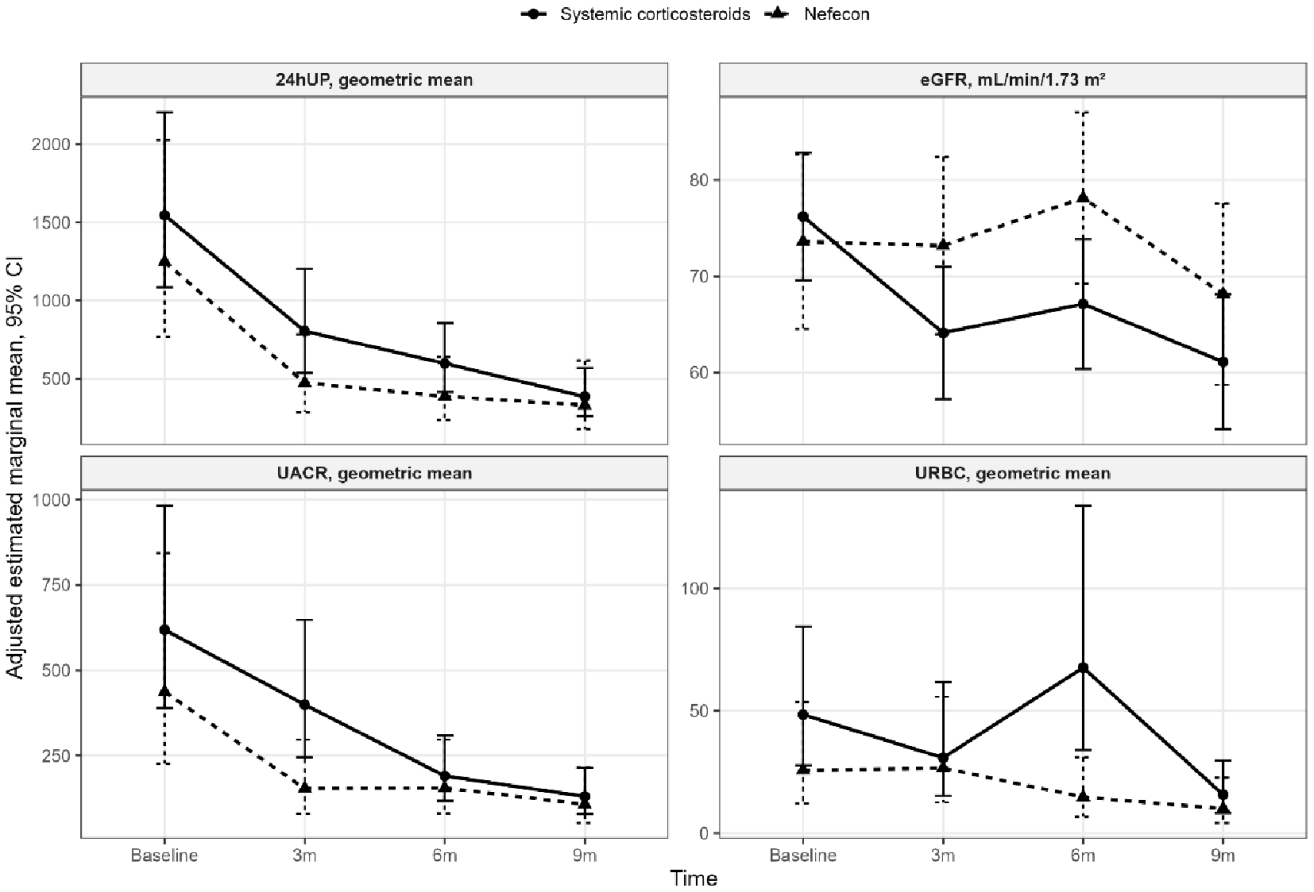
Longitudinal changes in renal outcomes over time.

After multiple imputation for missing data and pooling using Rubin’s rules, linear mixed-effects models were constructed to evaluate the longitudinal associations between treatment group and renal outcome trajectories. Models were specified with the systemic corticosteroid group at baseline as the reference, included fixed effects for treatment group, follow-up time, and their interaction, adjusted for a treatment group, time, their interaction, baseline eGFR, baseline log-transformed 24-hour urinary protein excretion, disease duration, CKD stage, Oxford MEST-C pathological variables, baseline use of ARB, SGLT2 inhibitors, and mineralocorticoid receptor antagonists, sex, age, systolic blood pressure, and diastolic blood pressure, and incorporated a random intercept for each participant to account for within-subject correlation (**Figure 2**).

As shown in **Table 1**, after further adjustment for baseline eGFR, baseline log-transformed 24hUP, disease duration, CKD stage, Oxford MEST-C score, baseline use of ARB, SGLT2 inhibitors, and MRA, as well as age, sex, systolic blood pressure, and diastolic blood pressure, no significant between-group differences were observed at baseline for eGFR, UACR, 24hUP, or URBC. The estimated baseline difference in eGFR between the Nefecon and systemic corticosteroid groups was attenuated and was not statistically significant (β = −2.61, 95% CI −13.90 to 8.69, P = 0.651). Similarly, baseline differences in UACR, 24hUP, and URBC were not statistically significant.

Time main effects indicated a significant decline in eGFR at 3, 6, and 9 months relative to baseline in the reference group (3 months: β = −12.08, 95% CI −19.85 to −4.31, P = 0.002; 6 months: β = −9.08, 95% CI −16.75 to −1.42, P = 0.020; 9 months: β = −15.09, 95% CI −23.06 to −7.13, P< 0.001). In contrast, proteinuria-related outcomes showed sustained improvement over time. UACR was significantly reduced at 3, 6, and 9 months compared with baseline, with corresponding geometric mean ratios of 0.65, 0.31, and 0.21, respectively. Similarly, 24hUP declined significantly at all follow-up time points, with geometric mean ratios of 0.52, 0.39, and 0.25 at 3, 6, and 9 months, respectively. URBC showed a significant reduction only at 9 months compared with baseline (β = −1.11, 95% CI −1.85 to −0.36, ratio = 0.33, P = 0.004).

With respect to treatment-by-time interaction effects, the Nefecon group showed a short-term, statistically significant favorable difference in eGFR at 6 months compared with the systemic corticosteroid group (β = 13.62, 95% CI 0.98 to 26.26, P = 0.035). However, the corresponding differences at 3 months (β = 11.67, 95% CI −1.34 to 24.68, P = 0.079) and 9 months (β = 9.64, 95% CI −3.07 to 22.34, P = 0.137) did not reach statistical significance. For UACR, the treatment-by-time interaction at 3 months suggested a trend toward greater early reduction in the Nefecon group, corresponding to a geometric mean ratio of 0.55, but this did not reach statistical significance after expanded adjustment (β = −0.61, 95% CI −1.27 to 0.05, P = 0.072). No significant treatment-by-time interactions were observed for UACR at 6 or 9 months, nor for 24hUP or URBC at any follow-up time point.

### 3.3 Adverse events

Adverse events observed during the follow-up period are summarized in **Table 3**. Overall, the incidence of infections and systemic corticosteroid-related adverse events was comparable or lower in the Nefecon group relative to the control group.

**Table 3:** Comparison of adverse events between Nefecon and control groups.

Adverse event	Control (%)	Nefecon (%)	OR (95% CI)	*P* ^a^
Mild infection (self-healing)	16	12.5	0.75 (0.21–2.73)	0.757
Moderate infection (medication required)	14	6.2	0.41 (0.08–2.11)	0.471
Severe infection (hospitalization)	12	0.0	–	0.077
Elevated blood sugar	4	3.1	0.77 (0.07–8.91)	1
Elevated blood pressure	6	0.0	–	0.277
Weight gain	6	0.0	–	0.277
Weight loss	2	6.2	3.27 (0.28–37.59)	0.557
Abdominal bloating/nausea	12	6.2	0.49 (0.09–2.59)	0.473
Gastrointestinal bleeding/ulcer	2	0.0	–	1
Muscle cramps/weakness	22	15.6	0.66 (0.20–2.11)	0.671
Acne	10	18.8	2.08 (0.58–7.48)	0.423
Itching	0	9.4	–	0.056
Cushingoid face	6	3.1	0.51 (0.05–5.08)	1
Insomnia	14	9.4	0.64 (0.15–2.66)	0.733
Dizziness/headache	12	0.0	–	0.077
Elevated eye pressure/blurred vision	8	3.1	0.37 (0.04–3.48)	0.644

^a^p values were derived from Fisher’s exact test unless otherwise indicated.

The frequencies of mild and moderate infections did not differ significantly between groups. Notably, severe infections requiring hospitalization occurred exclusively in the control group (12.0%), whereas no such events were observed in the Nefecon group, with the between-group difference approaching statistical significance (P = 0.077). In addition, classical systemic corticosteroid-related adverse events, including elevated blood pressure, weight gain, and cushingoid appearance, were observed only in the control group, although none of these comparisons reached statistical significance.

Other adverse events, including hyperglycemia, gastrointestinal discomfort, muscle cramps or weakness, acne, insomnia, dizziness, or headache, occurred at similar rates in the two groups. Overall, no safety signal suggesting an increased risk of adverse events associated with Nefecon treatment was identified. Given the modest sample size and limited event counts, these safety observations should be interpreted as descriptive and hypothesis-generating rather than confirmatory.

### 3.4 Sensitivity analyses

In sensitivity analyses using linear mixed-effects models with random slopes for time, treatment-by-time interaction effects were largely consistent with those observed in the primary random-intercept models (**Table 4**). For eGFR, the treatment-by-time effects at 3 and 6 months remained statistically significant under the random-slope specification, indicating robustness of the observed short- to mid-term differences in renal function trajectories. Similarly, treatment-by-time effects for UACR at 3 and 9 months were stable across random-effects structures. In contrast, no significant treatment-by-time interactions were observed for 24-hour urinary protein or urinary red blood cells in either model, suggesting robust null effects for these outcomes.

**Table 4:** Sensitivity analyses of treatment-by-time interaction effects using alternative random-effects structures.

Outcome	Time	Random intercept LMM β (95% CI)	P	Random slope LMM β (95% CI)	*P*
24hUP (log)	3 months	−0.41 (−1.05, 0.22)	0.199	−0.41 (−1.01, 0.18)	0.174
	6 months	−0.35 (−0.97, 0.27)	0.271	−0.35 (−0.98, 0.28)	0.279
	9 months	0.04 (−0.69, 0.76)	0.917	0.04 (−0.76, 0.83)	0.925
URBC (log)	3 months	1.15 (−0.35, 2.65)	0.133	1.15 (−0.35, 2.65)	0.133
	6 months	−0.28 (−1.91, 1.35)	0.738	−0.28 (−1.91, 1.35)	0.738
	9 months	0.79 (−0.78, 2.35)	0.324	0.79 (−0.78, 2.35)	0.325
eGFR	3 months	11.75 (−0.25, 23.75)	0.055	11.75 (1.60, 21.89)	0.023
	6 months	13.82 (2.01, 25.63)	0.022	13.82 (1.53, 26.12)	0.028
	9 months	10.74 (−1.20, 22.68)	0.078	10.74 (−4.83, 26.31)	0.176
UACR (log)	3 months	−0.70 (−1.32, −0.09)	0.024	−0.70 (−1.31, −0.10)	0.023
	6 months	0.19 (−0.48, 0.86)	0.574	0.19 (−0.48, 0.86)	0.576
	9 months	−0.72 (−1.38, −0.07)	0.031	−0.72 (−1.40, −0.05)	0.036

*β* estimates represent treatment × time interaction effects relative to the baseline control group. Models were fitted on multiply imputed datasets and adjusted for age, sex, SBP, and DBP. Random slope models additionally allowed subject-specific time trajectories.

Missingness was mainly concentrated in repeated follow-up laboratory measurements. The highest missing rates were observed for 24hUP at 3 and 9 months (both 34.1%), URBC at 3 months (32.9%), URBC at 9 months (29.3%), and UACR at 9 months (23.2%). In contrast, treatment group, sex, age, CKD stage, and during-treatment nephroprotective therapy variables were complete. Baseline eGFR had a low missing rate of 2.4%, baseline ARB/SGLT2 inhibitor/MRA use had missing rates of 4.9%, and Oxford MEST-C variables had missing rates of 11.0%. Complete-case sensitivity analyses yielded generally directionally consistent but less precise estimates compared with the multiple-imputation analyses. A detailed missingness summary is provided in **Supplementary Table 1**.

## 4 Discussion

This real-world study provides head-to-head comparative evidence on the effectiveness and safety of Nefecon versus systemic corticosteroids in patients with IgA nephropathy (IgAN). Most pivotal trials have enrolled selected IgAN populations under controlled conditions, whereas systemic glucocorticoids-despite decades of use-may yield heterogeneous benefits and can cause clinically relevant adverse effects, particularly at higher doses. In line with KDIGO recommendations, optimized supportive care (including renin–angiotensin–aldosterone system blockade for proteinuria control) remains foundational (2); however, supportive therapy alone is frequently insufficient for patients at higher risk of progression, prompting the use of immunomodulatory therapy in clinical practice. Against this background, our longitudinal analyses suggest that Nefecon and systemic corticosteroids both improve proteinuria-related outcomes over time. The primary outcome (24hUP) showed no significant between-group difference at any time point.After expanded adjustment, Nefecon showed a trend toward greater early albuminuria reduction and an exploratory short-term favorable eGFR difference at 6 months, but no sustained between-group difference was observed at 9 months. These eGFR findings should be interpreted with caution and are hypothesis-generating rather than confirmatory.

The NefIgArd trial demonstrated that targeted-release budesonide effectively reduces proteinuria and stabilizes kidney function in selected populations with IgA nephropathy (5). Nonetheless, randomized controlled trials are conducted under highly controlled conditions and may not fully reflect routine clinical practice, and direct comparative real-world evidence between Nefecon and systemic corticosteroids remains limited. In our study, both groups exhibited significant longitudinal reductions in UACR at 3, 6, and 9 months. After expanded adjustment, the treatment-by-time interaction for UACR at 3 months suggested a trend toward greater early reduction in the Nefecon group, but this did not reach statistical significance (*β* = −0.61, corresponding geometric mean ratio 0.55; *P* = 0.072). This early advantage is biologically plausible: since galactose-deficient IgA1 (Gd-IgA1) plays a central pathogenic role in IgAN, selectively targeting the distal ileum corresponding to Peyer’s patches may rapidly suppress mucosal immune activation and reduce upstream production of pathogenic IgA, thereby translating into a prompt albuminuria response. Consistently, Nefecon is designed to suppress gut-associated lymphoid tissue, particularly Peyer’s patches, which represent a major source of pathogenic galactose-deficient IgA1 (7). By contrast, systemic corticosteroids exert broader immunosuppressive effects and may require longer exposure to achieve comparable reductions in proteinuria.

Importantly, the between-group difference in UACR was not sustained at 6 and 9 months in our longitudinal models. This convergence may reflect that, over time, residual albuminuria increasingly represents chronic structural glomerular injury (e.g., podocyte and capillary wall damage) rather than predominantly immune-driven activity, thereby attenuating the differential impact of upstream mucosal immunomodulation. Regarding 24-hour urinary protein excretion, both treatments produced significant time-dependent reductions, yet no significant group-by-time interaction was detected at any follow-up point, suggesting broadly comparable antiproteinuric trajectories in terms of total protein excretion. Collectively, these findings support that Nefecon may provide an early albuminuria benefit while achieving overall antiproteinuric efficacy similar to systemic steroids over 9 months.

Hematuria, assessed by urinary red blood cell count, declined over time in both groups without a clear differential treatment effect in the interaction models. Emerging evidence indicates that persistent microscopic hematuria reflects ongoing glomerular inflammation and capillary wall injury in IgAN (8, 9). Mechanistically, hematuria is thought to arise from aberrant production of Gd-IgA1, which promotes the generation of anti-glycan IgG antibodies, leading to circulating immune complex formation, mesangial deposition, and subsequent renal injury (10). Nefecon, by selectively modulating gut-associated lymphoid tissue and reducing upstream Gd-IgA1 production, may plausibly attenuate inflammatory activity and facilitate restoration of capillary wall integrity, thereby reducing erythrocyte leakage into the urinary space (7). In our cohort, however, the absence of a robust interaction effect may be explained by baseline imbalance (lower baseline URBC in the targeted-release budesonide group) and the known short-term variability of uRBC measurements, for which time-averaged hematuria is often recommended to define persistent hematuria and assess prognostic implications. Prior studies have shown that remission of hematuria correlates with treatment response and improved renal survival (11) and that persistent hematuria is an independent risk factor for adverse outcomes (12), with higher degrees of persistent hematuria independently predicting worse prognosis (13). Additionally, early remission of persistent hematuria (within 6 months after kidney biopsy) has been associated with a reduced risk of kidney disease progression (14). Taken together, our hematuria findings should be interpreted as supportive but not definitive, and future studies incorporating time-averaged hematuria may better capture sustained inflammatory activity and treatment response.

Second, after expanded adjustment for baseline eGFR, baseline proteinuria, disease duration, CKD stage, Oxford MEST-C pathological variables, baseline nephroprotective therapy, and demographic and blood pressure covariates, Nefecon was associated with an exploratory short-term favorable difference in eGFR at 6 months compared with systemic corticosteroids. However, this difference was not statistically significant at 3 months or 9 months. Therefore, this finding should not be interpreted as evidence of sustained renal functional preservation or structural disease modification. Short-term eGFR changes over a 9-month follow-up period may be influenced by hemodynamic variation, steroid tapering, intercurrent clinical events, and optimization of supportive therapy. Longer follow-up is required to determine whether this short-term signal translates into durable renal protection in IgA nephropathy. Importantly, the pharmacokinetic characteristics of Nefecon support its potential tolerability advantages in patients with renal impairment: Nefecon is predominantly cleared via hepatic metabolism, and renal impairment is not expected to significantly affect drug metabolism (15).

Systemic glucocorticoid therapy in IgAN remains controversial because not all patients benefit and severe side effects can occur, particularly with high-dose regimens. In our study, adverse events differed between treatments: Severe infections occurred more frequently in patients receiving systemic corticosteroids; these safety findings should be interpreted cautiously given the modest sample size and limited event numbers. Although several comparisons were limited by sparse events, the overall pattern aligns with prior reports emphasizing the safety advantage of targeted delivery and the emergence of novel adverse events requiring pharmacovigilance (15). Clinically, these findings support considering Nefecon as an alternative for patients who are poor candidates for prolonged systemic steroid exposure due to infection risk or metabolic vulnerability. Although severe infections were observed only in the systemic corticosteroid group, the difference did not reach statistical significance, and the small number of events precludes definitive conclusions. These safety findings are best viewed as hypothesis-generating and require confirmation in larger studies with longer follow-up.

This study has several limitations inherent to its observational design. First, the sample size is modest, with only 32 patients in the Nefecon group and 50 in the systemic corticosteroid group, which limits statistical power to detect smaller but potentially clinically meaningful differences, particularly for secondary outcomes and subgroup analyses, and increases the risk of overfitting in the expanded mixed-effects model. Although covariates were selected *a priori* based on clinical relevance and sequential models demonstrated stable effect estimates (**Supplementary Table 3**), the potential for overfitting cannot be fully excluded. Second, the follow-up duration is limited to 9 months, which is insufficient to assess long-term renal function preservation or disease modification; longer follow-up is needed to determine whether the short-term signals observed translate into durable clinical benefits. Third, although we adjusted for baseline use of ARB, SGLT2 inhibitors, and MRA in the mixed-effects models, substantial baseline differences in these nephroprotective therapies existed between groups-particularly in ARB use (67.9% vs. 14.0%), SGLT2 inhibitor use (35.7% vs. 2.0%), Oxford C score, and baseline eGFR-and regression adjustment cannot fully eliminate the potential for residual confounding due to unmeasured or imperfectly measured covariates. The limited sample size of this real-world cohort precluded propensity score matching or weighting, as such approaches would have substantially reduced statistical power. Therefore, our findings should be interpreted with particular caution when considering the comparative effectiveness of the two treatments, and causal inference cannot be drawn from this observational comparison. Fourth, we did not perform propensity score matching or weighting, which could have further balanced baseline covariates; this represents an additional methodological limitation, and future studies with larger sample sizes may consider propensity-based approaches. Fifth, as with all non-randomized observational studies, residual confounding remains highly likely despite our efforts to adjust for a wide range of measured covariates. Unmeasured confounders-such as frailty, adherence, socioeconomic status, and detailed dietary or lifestyle factors-may still influence the observed associations. Therefore, causal inference cannot be drawn from this study, and our findings should be interpreted as hypothesis-generating rather than confirmatory. Missing data represent another limitation; missingness was mainly concentrated in follow-up laboratory measurements, particularly 24hUP and URBC, whereas treatment assignment and core baseline variables were largely complete. We used multiple imputation under the MAR assumption and included extensive baseline, treatment, and longitudinal outcome variables in the imputation model to improve its plausibility. However, the MAR assumption cannot be formally verified, and missing-not-at-random mechanisms remain possible in this retrospective real-world cohort. Complete-case analyses showed wider confidence intervals and lower precision, particularly for eGFR, further supporting cautious interpretation of the exploratory short-term renal function findings. In addition, our study did not measure Gd-IgA1, anti-glycan antibodies, or complement activation markers. Therefore, our mechanistic interpretation is based on existing literature rather than direct immunological measurements from this cohort.

In summary, the primary outcome of this study-24-hour proteinuria-did not differ significantly between the Nefecon and systemic corticosteroid groups. The exploratory short-term eGFR signal at 6 months, while intriguing, was not sustained and requires confirmation in larger studies with longer follow-up. Thus, our findings do not support a claim of superior renal protection with Nefecon over systemic corticosteroids, but rather suggest that Nefecon may be a reasonable alternative with comparable antiproteinuric efficacy and potentially better tolerability.

In conclusion, in this real-world cohort with limited sample size and 9-month follow-up, Nefecon demonstrated antiproteinuric efficacy broadly comparable to systemic corticosteroids, but the primary outcome (24hUP) did not differ significantly between groups.After expanded adjustment, Nefecon showed a trend toward greater early albuminuria reduction and an exploratory short-term favorable eGFR difference at 6 months; however, this eGFR difference was not sustained at 9 months and should not be interpreted as evidence of sustained renal functional preservation or structural disease modification. Larger studies with longer follow-up are needed to confirm the durability and clinical significance of these findings.

## Data Availability

The original contributions presented in the study are included in the article/**Supplementary Material**. Further inquiries can be directed to the corresponding authors.
