# Supplementary material for: Targeted-release budesonide versus systemic corticosteroids in IgA nephropathy: a real-world comparative study
Source: Front Immunol. 2026 Jul 29;17:1831633. doi: 10.3389/fimmu.2026.1831633 (PMC13461704; doi:10.3389/fimmu.2026.1831633)
Supplement: Supplementary file 1 [file Supplementaryfile1.docx]

**Supplementary Table S1**: Missing data summary

| **Variable category** | **Variable** | **Missing, n (%)** |
| --- | --- | --- |
| Core baseline variables | group | 0 (0.0) |
| Core baseline variables | sex | 0 (0.0) |
| Core baseline variables | age | 0 (0.0) |
| Baseline renal function | baseline eGFR | 2 (2.4) |
| Baseline supportive therapy | BARB | 4 (4.9) |
| Pathology | Oxford M/E/S/T/C | 9 (11.0) |
| Follow-up outcomes | 3-month 24hUP | 28 (34.1) |
| Follow-up outcomes | 9-month 24hUP | 28 (34.1) |
| Follow-up outcomes | 3-month URBC | 27 (32.9) |
| Follow-up outcomes | 9-month URBC | 24 (29.3) |
| Follow-up outcomes | 9-month UACR | 19 (23.2) |

**Supplementary Table 2. Sensitivity analysis excluding patients with CKD stage 5 at baseline**

| **Outcome** | **Time point** | **Term** | **Estimate** | **SE** | **95% CI** | **P** | **Transformed effect** |
| --- | --- | --- | --- | --- | --- | --- | --- |
| eGFR | 3 months | groupNewDrug:time3m | 13.00 | 6.29 | 0.68 to 25.33 | 0.039 | — |
| eGFR | 6 months | groupNewDrug:time6m | 15.57 | 6.25 | 3.32 to 27.82 | 0.013 | — |
| eGFR | 9 months | groupNewDrug:time9m | 12.33 | 6.32 | -0.05 to 24.72 | 0.051 | — |
| log(24hUP) | 3 months | groupNewDrug:time3m | -0.38 | 0.33 | -1.03 to 0.28 | 0.258 | Ratio = 0.68, 95% CI: 0.36 to 1.32 |
| log(24hUP) | 6 months | groupNewDrug:time6m | -0.26 | 0.34 | -0.92 to 0.40 | 0.444 | Ratio = 0.77, 95% CI: 0.40 to 1.50 |
| log(24hUP) | 9 months | groupNewDrug:time9m | 0.07 | 0.35 | -0.61 to 0.75 | 0.835 | Ratio = 1.07, 95% CI: 0.54 to 2.12 |
| log(UACR) | 3 months | groupNewDrug:time3m | -0.61 | 0.35 | -1.30 to 0.07 | 0.078 | Ratio = 0.54, 95% CI: 0.27 to 1.07 |
| log(UACR) | 6 months | groupNewDrug:time6m | 0.16 | 0.36 | -0.54 to 0.86 | 0.658 | Ratio = 1.17, 95% CI: 0.58 to 2.35 |
| log(UACR) | 9 months | groupNewDrug:time9m | 0.13 | 0.38 | -0.61 to 0.88 | 0.726 | Ratio = 1.14, 95% CI: 0.54 to 2.40 |
| log(URBC) | 3 months | groupNewDrug:time3m | 0.41 | 0.57 | -0.70 to 1.52 | 0.471 | Ratio = 1.50, 95% CI: 0.50 to 4.57 |
| log(URBC) | 6 months | groupNewDrug:time6m | -0.81 | 0.59 | -1.97 to 0.36 | 0.174 | Ratio = 0.45, 95% CI: 0.14 to 1.43 |
| log(URBC) | 9 months | groupNewDrug:time9m | 0.06 | 0.57 | -1.06 to 1.19 | 0.911 | Ratio = 1.07, 95% CI: 0.35 to 3.28 |

Abbreviations: eGFR, estimated glomerular filtration rate; UACR, urine albumin-to-creatinine ratio; 24hUP, 24-hour urinary protein; URBC, urinary red blood cell count; SE, standard error; CI, confidence interval. For eGFR, the estimate represents the between-group difference in change from baseline at each time point. For log-transformed outcomes, the transformed effect is shown as the ratio of ratios after exponentiation.

**Supplementary Table 3. Sequential models for the between-group difference in eGFR change from baseline to 6 months**

| **Model** | **Adjustment covariates** | ***β*** | **SE** | **95% CI** | **P** | **No. of adjusted covariates** | **Imputed datasets used** | **N** |
| --- | --- | --- | --- | --- | --- | --- | --- | --- |
| Model 1 | Unadjusted | 14.47 | 8.25 | -1.70 to 30.64 | 0.079 | 0 | 50 | 82 |
| Model 2 | Age, sex, SBP, DBP | 10.69 | 7.60 | -4.21 to 25.60 | 0.160 | 4 | 50 | 82 |
| Model 3 | Model 2 + baseline eGFR and baseline log-transformed 24hUP | 7.18 | 7.02 | -6.57 to 20.93 | 0.306 | 6 | 50 | 82 |
| Model 4 | Model 3 + disease duration, baseline CKD stage, Oxford MEST-C lesions | 10.00 | 8.47 | -6.62 to 26.61 | 0.238 | 13 | 50 | 82 |
| Model 5 | Model 4 + baseline ARB, SGLT2 inhibitor, and MRA use | 18.60 | 10.76 | -2.49 to 39.69 | 0.084 | 16 | 50 | 82 |

Abbreviations: eGFR, estimated glomerular filtration rate; SBP, systolic blood pressure; DBP, diastolic blood pressure; 24hUP, 24-hour urinary protein; CKD, chronic kidney disease; MEST-C, Oxford classification lesions; ARB, angiotensin receptor blocker; SGLT2, sodium-glucose cotransporter 2; MRA, mineralocorticoid receptor antagonist; SE, standard error; CI, confidence interval. The β estimate represents the between-group difference in eGFR change from baseline to 6 months for Nefecon compared with systemic corticosteroids.
